# Adenylation Domain Engineering Enables Biosynthesis of an Improved Polymyxin Antibiotic

**DOI:** 10.1002/anie.9880949

**Published:** 2026-09-09

**Authors:** Maximilian Müll, Pia Lanvers, Giulia Ravagnan, Kilian Klaus, Olena Nosovska, Ivan Vilotijevic, Jochen Schmid, Hajo Kries

**Affiliations:** ^1^ Department of Technical Biochemistry University of Stuttgart Stuttgart Germany; ^2^ Biosynthetic Design of Natural Products Leibniz Institute for Natural Product Research and Infection Biology (HKI) Jena Germany; ^3^ Institute of Molecular Microbiology and Biotechnology University of Münster Münster Germany; ^4^ Institute of Organic Chemistry and Macromolecular Chemistry Friedrich Schiller University Jena Jena Germany

**Keywords:** antibiotics, biochemistry, biosynthesis, fatty acid, mutagenesis, natural product, nonribosomal peptide, *Paenibacillus polymyxa*, polymyxin

## Abstract

Polymyxins are potent nonribosomal peptide antibiotics, yet their clinical use is limited by a narrow therapeutic window and nephro‐/neurotoxicity. Conventional efforts to improve these agents rely on the incorporation of nonnatural building blocks that cannot be accessed biosynthetically and therefore must be introduced by total synthesis. Here we report a biosynthetic route to a next‐generation polymyxin that circumvents this limitation. By engineering the substrate‐binding pocket of a nonribosomal adenylation (A‐)domain, we created a mutationally expanded pocket that selectively activates the fatty amino acid l‐2‐aminodecanoic acid (l‐Ada). Incorporation of this engineered A‐domain into the native polymyxin biosynthesis enabled production of Ada‐polymyxin with high potential for lowered toxicity directly in *Paenibacillus polymyxa* fermentation. This work demonstrates that rational redesign of nonribosomal A‐domains can redirect natural product biosynthesis toward chemically diverse, therapeutically superior antibiotics, opening new avenues for combating drug‐resistant bacteria.

## Graphical abstract

To enable incorporation of l‐2‐aminodecanoic acid into polymyxin by fermentation, a single Trp‐to‐Ala substitution is rationally introduced into the adenylation‐domain residue of its nonribosomal peptide synthetase (NRPS) and efficiently switches substrate selectivity. The engineered strain produces biologically active Ada‐polymyxin, demonstrating that NRPS engineering provides a direct biosynthetic route to a nonnatural polymyxin with potential therapeutic advantages.

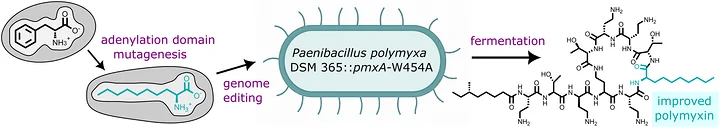

Natural products remain a primary source of therapeutics, yet structural tweaks are often needed to improve potency, safety, or pharmacokinetics [1, 2]. Illustrative cases include double‐bond insertion in voclosporin reducing side effects [3] (Figure 1A), esterification with pivaloyloxymethyl in tebipenem pivoxil boosting oral bioavailability [4], and hydrogen‐to‐fluorine exchange in flurithromycin conferring acid stability [5]. Total synthesis offers structural flexibility but is resource‐intensive, whereas biosynthesis followed by chemical modification is more economical but structurally limited. Engineered biosynthesis has emerged as an attractive route to structurally diverse molecules, including architectures inaccessible by post‐biosynthetic chemical modification [6, 7, 8, 9, 10, 11, 12].

**FIGURE 1:**
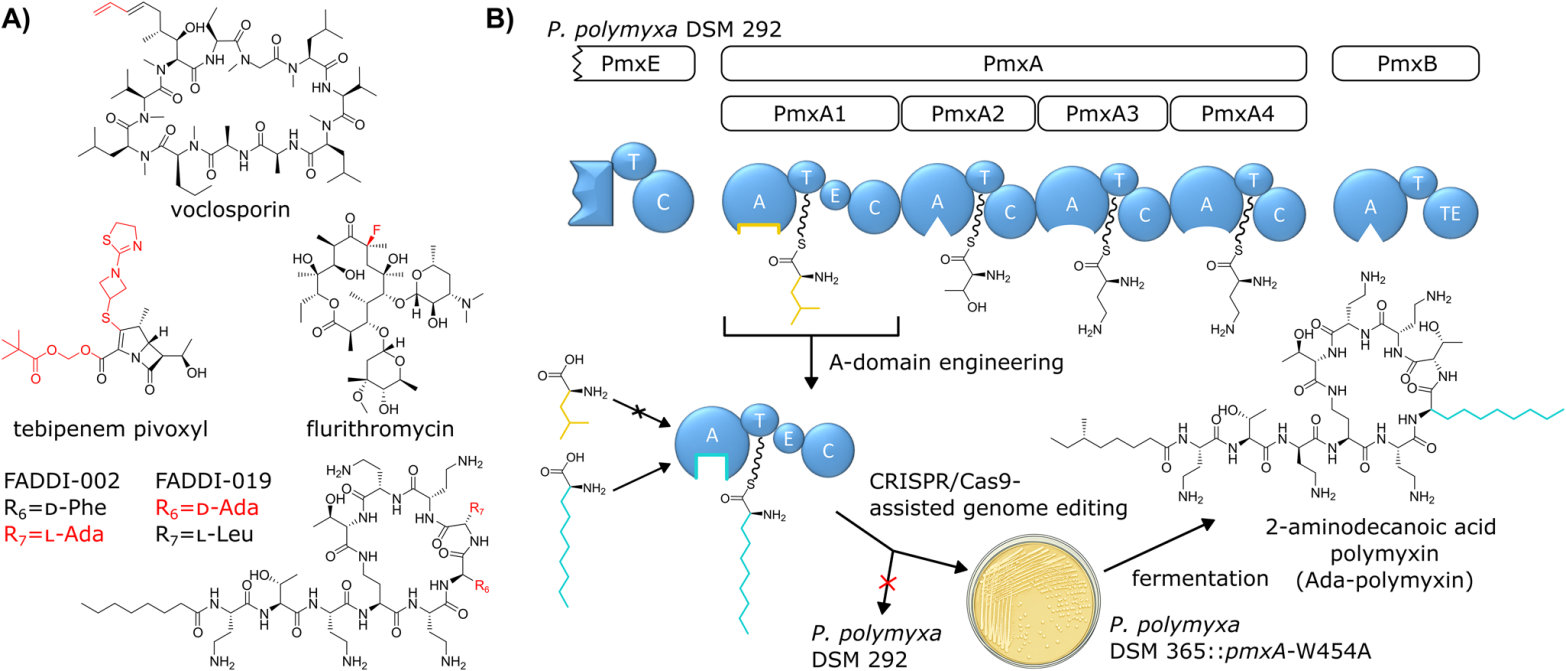
(A) Drugs derived from natural products by chemical synthesis with modifications in red. (B) Strategy for the biosynthetic production of Ada‐polymyxin. Mutation of the A‐domain of PmxA1 in the polymyxin synthetase of *P. polymyxa* DSM 292 generated a variant selective for Ada. The mutation was introduced into the genome of the producing strain DSM 365, because genome editing failed with DSM 292 and Ada‐polymyxin was obtained by fermentation.

Among natural product‐based drugs, polymyxins are key “last‐line” antibiotics [13]. They comprise a diverse class of lipoylated peptides with a linear tripeptide and a heptapeptide ring, though clinical use is limited to polymyxin B and polymyxin E (colistin) [14] despite numerous subclasses [15]. These compounds are biosynthesized by nonribosomal peptide synthetases (NRPSs) and produced industrially via fermentation of *Paenibacillus polymyxa* [16]. However, their medical use is limited by acute nephrotoxicity and spreading resistance due to agricultural overuse [17, 18, 19]. Efforts to discover new polymyxins have focused on modifying toxicity and activity spectra, often through total synthesis [20, 21, 22, 23]. For instance, chemical peptide synthesis inspired by genome analysis has led to the discovery of a new polymyxin‐like, resistance‐evading molecule called macolacin [24]. Analogues FADDI‐002 and FADDI‐019 (Figure 1A) developed by Velkov et al. [22] containing 2‐aminodecanoic acid (Ada) have overcome polymyxin resistance by increasing the hydrophobicity at positions 6 and 7. Those compounds regained activity against clinical isolates of resistant *A. baumannii* strains and were tolerated well in mouse thigh models, but have not been biosynthetically accessible.

An ideal route to improved polymyxins would harness the NRPS assembly line of a producing organism re‐engineered to incorporate nonnatural residues. NRPSs are large, modular enzymes [25] in which each module typically contains an A‐domain for substrate selection, a thiolation domain (T‐domain) for carrier‐protein attachment, and a condensation domain (C‐domain) for peptide bond formation. This modularity makes NRPSs attractive targets for engineering [26], and recent advances in module recombination [8] have heightened the demand for A‐domains that can activate noncanonical amino acids. Although considerable progress has been made in reprogramming A‐domain specificity [6, 7, 11, 27, 28, 29, 30, 31, 32, 33], creating fully functional, engineered NRPS pathways for new peptide antibiotics remains challenging.

To provide a biosynthetic route toward Ada‐polymyxin, we generated an A‐domain mutant capable of specifically adenylating Ada and related amino acids through rational site‐directed mutagenesis. Then, we incorporated the A‐domain mutation into the genome of *P. polymyxa* DSM 365, and produced Ada‐polymyxin [22] (Figure 1B), which is a close structural analogue of resistance‐overcoming bis‐lipopetide FADDI‐019.

To enable Ada incorporation, we first examined the NRPS module PmxA_1_ from *P. polymyxa* DSM 292 [34], which installs the sixth residue of polymyxin M [35] (identified by antiSMASH [36], Figure S1). The natural amino acid substrate of PmxA_1_ was predicted to be l‐Leu. We cloned the ATEC‐encoding fragment of *pmxA_1_*, including the epimerization (E‐)domain that inverts stereochemistry, into *E. coli* HM0079 [37], expressed it heterologously, and purified the protein (Figure S2). A truncated C‐domain upstream of the A‐domain appears inactive. Adenylation specificity was assessed with a MesG/hydroxylamine‐based pyrophosphate release assay [38]. The wild type A‐domain efficiently activated l‐Leu, confirming the antiSMASH prediction (Figure 2A), whereas no activity was observed with the target substrate l‐Ada. These results establish the baseline specificity of PmxA_1_ and provide a platform for subsequent A‐domain engineering to accept Ada.

**FIGURE 2:**
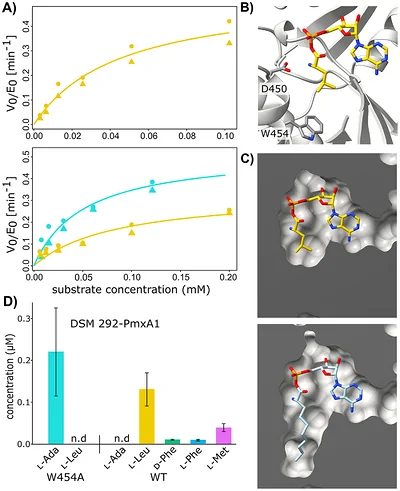
Module PmxA1‐W454A from strain DSM 292 shows l‐Ada specificity. (A) Michaelis–Menten kinetics for the wild type (top) with l‐Leu (yellow line). Activity for l‐Ada is not detectable. W454A mutant (bottom) with l‐Leu (yellow) and l‐Ada (cyan). Replicates measured with two different batches of protein are labelled with circles and triangles. (B) Model of the PmxA1‐A active site with l‐Leu‐AMP as ligand. (C) Cut‐through representation of models of the wild type bound to l‐Leu‐AMP (top) and the W454A variant bound to l‐Ada‐AMP (bottom), illustrating how the W454A mutation makes space in the active site for the larger ligand. (D) HAMA specificity profiles of the W454A variant and the wild type of module PmxA1 from strain DSM 292. HAMA was performed with all proteinogenic amino acids, d‐Phe, d‐Val, and l‐Ada in competition. All substrates omitted from the plot yielded no detectable hydroxamates. Low levels of d/l‐Phe HA and Met HA were observed for the wild type, but below the limit of quantification. Reactions were run as biological triplicates with independently purified batches of protein. n.d = not detectable.

To evaluate whether the A‐domain of PmxA_1_ can be retargeted to l‐Ada, we generated a homology model with the Chai‐1 [39] pipeline (Figure 2B). The substrate‐bound model reveals that the bulky side chain of l‐Ada sterically clashes with the pocket, precluding adenylation. The active site bottom is capped by Trp454 (Figure 2B,C), a residue conserved in the A‐domains of the Bacillial NRPSs GrsA (gramicidin S) and TycA (tyrocidine), which naturally activate phenylalanine (Phe). Prior engineering of these Trp residues to Ser successfully switched specificity from Phe to larger, clickable aryl amino acids (e.g., propargyloxy‐Phe) [6, 10, 40]. This precedent suggests that mutating Trp454 to a smaller side chain could open the pocket sufficiently to accommodate l‐Ada, providing a rational route for reprogramming PmxA_1_ specificity.

To overcome the steric clash that prevents l‐Ada activation, we replaced the bulky Trp454 in PmxA_1_ with a small residue. Alanine was chosen (W454A) to retain a hydrophobic environment compatible with the Ada side chain. Michaelis–Menten parameters of adenylation were determined for the W454A mutant and compared to the wild type enzyme (Figure 2A). Gratifyingly, we achieved flipped selectivity for the fatty amino acid l‐Ada over l‐Leu with the large‐to‐small mutation W454A. The catalytic efficiency (*k*_cat_/*K*_M_) of the W454A variant with l‐Ada is 10 ± 2 mM^−1^ min^−1^, which is comparable to the wild type with l‐Leu as substrate (11.9 ± 1.6 mM^−1^ min^−1^). While activity was not detectable for l‐Ada with the wild type, the W454A variant still has reduced activity for l‐Leu with a *k*_cat_/*K*_M_ of 4 ± 2 mM^−1^ min^−1^. When alternative chain lengths were offered to the variant, a higher *k*_cat_/*K*_M_ was observed for the longer‐chain fatty amino acid l‐2‐aminododecanoic acid (40 ± 10 mM^−1^ min^−1^, Figure S3). The shorter‐chain l‐2‐aminooctanoic acid was activated only by the wild type with a *k*_cat_/*K*_M_ of 19 ± 3 mM^−1^ min^−1^, being slightly preferred over l‐Leu.

To probe A‐domain multispecificity, we applied the multiplexed hydroxamate assay (HAMA) [41], which traps transient aminoacyl adenylates as stable hydroxamates (HAs) via hydroxylamine quenching. The resulting HAs are quantified by LC‐MS/MS using targeted multiple reaction monitoring (Figure 2D); because multiple amino acids compete in a single reaction, the product distribution directly reflects the specificity constants (*k*_cat_/*K*_M_). A custom Ada HA standard was prepared from Fmoc Ada acid by methyl ester formation (TMSCl, MeOH) followed by hydroxylamine conversion (Figure S4). HAMA profiling revealed predominant Leu HA formation with wild type PmxA_1_ and exclusive Ada HA formation with the W454A mutant, confirming the engineered specificity switch.

A Boltz‑2 homology model of the W454A A‑domain complexed with l‑Ada‑AMP (Figure 2C) was generated and subjected to 100 ns of molecular‑dynamics simulation in the CHARMM36 force field (Figures S5 and S6). The enlarged pocket comfortably accommodates the Ada side chain, yielding a stable binding pose throughout the trajectory. In contrast, the wild type pocket forces the Ada side chain into the T‑domain channel, adopting a bent conformation that is likely incompatible with T‑domain loading and adenylation. Although computational modeling suggests that the wild type enzyme can bind l‑Ada, the distorted conformation might explain the lack of activity observed experimentally.

A comparative analysis shows that the W454A variant aligns closely with a Chai‑1 model of the putative Ada‑activating GraA_5_ domain from *Fusarium graminearum* [42] (RMSD  =  0.9 Å; Figure S7). Both enzymes share a “small‑hydrophobic” specificity code [43, 44] (PmxA_1_‑A‑W454A: DAAIVAIVL; GraA_5_: DVGVVGVLA), differing only at the conserved Asp, underscoring the relevance of the W454A substitution for accommodating bulky fatty‑acid substrates.

After reprogramming PmxA_1_‑A to accept Ada, we attempted to generate a dipeptide by pairing module 6 (PmxA_1_) with module 7 (PmxA_2_, Thr‑specific). Both wild type PmxA_1‑2_ and the W454A variant expressed well (Figure S2), but peptide‑formation assays yielded no detectable product, likely because the excised modules cannot initiate synthesis without the native lipoylated hexapeptide intermediate.

To produce the modified polymyxin through fermentation, it was initially attempted to integrate mutation *pmxA*‐W454A into the genome of *P. polymyxa* DSM 292. For this purpose, the genome of *P. polymyxa* DSM 292 was sequenced. However, cloning turned out to be challenging and *P. polymyxa* DSM 365 was used instead, as the CRISPR‐Cas9 genome editing system developed by Rütering et al. [45] works efficiently in this strain. Comparison of the polymyxin biosynthetic gene clusters of DSM 365 and DSM 292 showed a sequence similarity of 96.1%. AntiSMASH analysis of the DSM 365 genome revealed a potential biosynthetic gene cluster for polymyxin P, exploitable for engineering. In this cluster, CRISPR‐Cas9 genome editing scarlessly introduced the desired mutation without any off‐target effects, which was confirmed by sequencing (Figure S8).

To prepare for biosynthesis of Ada‐polymyxin, we tested and optimized DSM 365 for its capability to produce native polymyxin P. A parent mass and a characteristic fragment that were, by comparison with a polymyxin B1 standard (Figure S9), consistent with polymyxin P1 (C_56_H_98_N_16_O_13_, *m*/*z* 596 [M+2H]^2+^ → 101) was detected in culture supernatants by triple quad LC‐MS/MS. To optimize production, different media compositions were tested containing different carbon sources and trace elements. Polymyxin P1 titres were measured with LC‐MS/MS after 50 h of fermentation. The best performing media (PBP Glucose) resulted in a polymyxin P1 production titre of 165 ± 3 µM (Table S1), and after partial purification, the identity of polymyxin P1 was confirmed by Orbitrap high‐resolution mass spectrometry (HRMS, Figure S1). To confirm the mutational effect, we heterologously produced PmxA1 and PmxA1‐W454A (Figure S2). HAMA profiling revealed the expected switch in specificity toward l‐Ada with the mutant (Figure 3A). Surprisingly, wild type PmxA1 showed specificity for d‐Phe and not for l‐Phe although an E‐domain is present.

**FIGURE 3:**
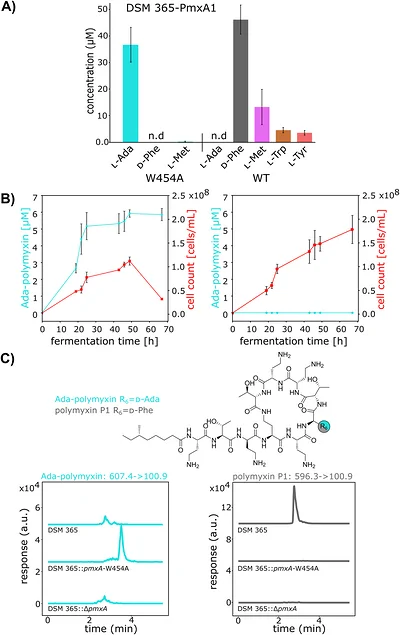
(A) HAMA specificity profiles of the W454A variant and the wild type of module PmxA1 from strain DSM 365. Reactions were run as biological duplicates with independently purified batches of protein. n.d = not detectable. (B) Time course of Ada‐polymyxin production and cell growth of DSM 365::*pmxA*‐W454A (left) and DSM 365::Δ*pmxA* (right). Ada‐polymyxin was quantified by UPLC‐MS/MS by comparison with a polymyxin B1 standard. (C) UPLC‐MS/MS analysis of Ada‐polymyxin (left) and polymyxin P1 production (right) through fermentation with strain DSM 365, DSM 365::*pmxA*‐W454A, and DSM 365::Δ*pmxA* (negative control). Samples taken after 3d of fermentation in the presence of 1 mM l‐Ada.

Then, the PBP Glucose media was supplemented with l‐Ada and employed for the biosynthesis of Ada‐polymyxin. Gratifyingly, DSM 365::*pmxA*‐W454A produces Ada‐polymyxin while the Δ*pmxA* control and the wild type do not (Figure 3B,C). Assuming identical LC‐MS/MS responses for Ada‐polymyxin and the polymyxin B1 standard, the production titre of Ada‐polymyxin is 6.3 ± 0.3 µM (Figure 3C). Additional confirmation of Ada‐polymyxin identity was provided through HRMS (Figure S10). Notably, cultures of DSM 365::*pmxA*‐W454A displayed cell lysis and a decline in viable counts after 50 h (Figure S11), whereas the Δ*pmxA* strain continued to grow (Figure 3B), indicating self‐intoxication at the observed concentration. Antimicrobial activity was assessed by disk diffusion assays against *E. coli* BL21. Extracts from the engineered strain generated an inhibition zone of 2.4  ±  0.5 cm, comparable to that produced by 0.6 µM polymyxin B1 (1.9  ±  0.2 cm); no zones were observed for the ΔpmxA control or for cultures lacking Ada (Figure S12). These results confirm that the secreted compound is biologically active Ada‐polymyxin.

We demonstrate that a single, rational substitution (W454A) in the PmxA_1_‐A‐domain reliably flips substrate selectivity from Leu or Phe to the fatty amino acid Ada with catalytic efficiency comparable to the native reaction, and that this engineered specificity can be implemented in vivo to produce Ada‑polymyxin by fermentation. The success mirrors earlier Trp substitutions in homologous binding pocket positions [6, 40] (e.g. GrsA‑W239S) and indicates a broadly applicable NRPS engineering strategy of replacing bulky pocket residues to admit larger noncanonical building blocks. A snug fit of the enzyme pocket to the extended alkyl chain, as indicated by structural modelling, explains the clean specificity switch. It is convenient for biosynthetic engineering that bulky, hydrophobic amino acids such as O‐propargyl‐Tyr or Ada occupy a niche of physicochemical properties that allows for excellent substrate specificity with the engineered substrates.

A 6 µM product titre provides a solid foundation for further strain improvement. Future work will focus on (i) endogenous Ada biosynthesis by transferring the putative gramillin type Ada pathway [42], eliminating the need for feeding; (ii) enhancing producer resistance to avoid self‐intoxication; and (iii) optimizing downstream domains. Collectively, these steps should raise yields and permit systematic Ada incorporation at additional positions. Our study demonstrates that A‐domain engineering is a practical route to generate medicinally relevant nonribosomal peptide analogues.

## Author Contributions

**Maximilian Müll**: conceptualization, investigation, writing – original draft, visualization, formal analysis, writing – review and editing, and methodology. **Pia Lanvers**: investigation, writing – review and editing, and visualization. **Giulia Ravagnan**: writing – review and editing, investigation, and visualization. **Kilian Klaus**: writing – review and editing, visualization, and investigation. **Olena Nosovska**: investigation and writing – review and editing. **Ivan Vilotijevic**: supervision, resources, project administration, and conceptualization. **Jochen Schmid**: project administration, supervision, resources, and conceptualization. **Hajo Kries**: funding acquisition, supervision, resources, project administration, writing – original draft, writing – review and editing, conceptualization, visualization, methodology, and formal analysis.

## Conflicts of Interest

The authors declare no conflicts of interest.

## Supporting information




**Supporting File**: The Supporting Information contains additional methods, tables, figures, and references [6, 27, 36, 37, 38, 41, 45, 46, 47, 48, 49, 50, 51, 52, 53, 54, 55, 56, 57, 58, 59, 60, 61, 62, 63, 64, 65, 66, 67, 68, 69].

## Data Availability

The data that supports the findings of this study are available in the Supporting Information of this article.
