# Supplementary material for: Adenylation Domain Engineering Enables Biosynthesis of an Improved Polymyxin Antibiotic
Source: Angew Chem Int Ed Engl. 2026 Sep 9;65(43):e9880949. doi: 10.1002/anie.9880949 (PMC13676401; doi:10.1002/anie.9880949)
Supplement: Supplementary file 1 [file ANIE-65-e9880949-s001.docx]

Supplementary for

Adenylation domain engineering enables
biosynthesis of improved polymyxin antibiotic

**M. Müll**^a,b^**; P. Lanvers**^c^**; G. Ravagnan**^c^**; K. Klaus**^a^**; O. Nosovska**^d^**; I. Vilotijevic**^d^**; J. Schmid**^c^ **; H. Kries**^a,b*^

^a^ Department of Technical Biochemistry, University of Stuttgart, Allmandring 31, 70569 Stuttgart, Germany

^b^ Biosynthetic Design of Natural Products, Leibniz Institute for Natural Product Research and Infection Biology (Hans Knöll Institute), Beutenbergstr. 11a, 07745 Jena, Germany

^c^ Institute of Molecular Microbiology and Biotechnology, University of Münster, Corrensstr. 3, 48149 Münster, Germany

^d^ Institute of Organic Chemistry and Macromolecular Chemistry, Friedrich Schiller University Jena, Humboldtstr. 10, 07743 Jena, Germany

[*hajo.kries@ibc.uni-stuttgart.de](mailto:*hajo.kries@ibc.uni-stuttgart.de)

Table of Contents

[Supplementary Materials and Methods	3](#_Toc237615049)

[AntiSMASH prediction	3](#_Toc237615050)

[Modelling and alignment of GraA_5_ and PmxA_1_-A-W454A	3](#_Toc237615051)

[General cloning	3](#_Toc237615052)

[Protein expression and purification	3](#_Toc237615053)

[MesG/hydroxylamine kinetics	4](#_Toc237615054)

[Molecular dynamics simulation	4](#_Toc237615055)

[HAMA specificity profiling	5](#_Toc237615056)

[Synthesis	5](#_Toc237615057)

[Bacterial strains	7](#_Toc237615058)

[Strain engineering	7](#_Toc237615059)

[Genome sequencing of *P. polymyxa* DSM 292	7](#_Toc237615060)

[Fermentation of polymyxins	8](#_Toc237615061)

[Quantification of polymyxins	8](#_Toc237615062)

[HRMS measurement	8](#_Toc237615063)

[Disk diffusion assay	9](#_Toc237615064)

[Supplementary Tables	10](#_Toc237615065)

[Supplementary Figures	17](#_Toc237615066)

[Bibliography	29](#_Toc237615067)

# Supplementary Materials and Methods

## AntiSMASH prediction

The genome of DSM 292 with the accession number
GCF_900406265.2 was uploaded at antiSMASH bacterial version web browser. The search was run with relaxed settings.

## Modelling and alignment of GraA_5_ and PmxA_1_-A-W454A

The sequence for each protein was pasted into the “protein” section of the Chai-1 prediction webserver <https://www.chaidiscovery.com/>. The SMILES for l-Ada-AMP were pasted into the “ligand” section. The predicted structures were loaded into PyMOL and PmxA_1_-A-W454A was aligned against GraA_5_.

## General cloning

In-Fusion cloning was performed following the supplier’s instructions. Primers for PCR amplification of inserts were designed with 15-20 bp long overlaps complementary to the insertion position in the vector DNA and ordered as synthetic oligonucleotides (Eurofins GmbH). Competent cells were transformed by heat shock. The insert was checked by Sanger sequencing (Genewiz). Restriction enzymes for linearization of vectors were purchased from New England Biolabs (NEB). PCR reactions were performed with Q5 High-Fidelity polymerase (NEB).

## Protein expression and purification

Proteins were expressed and purified as described previously with minor modifications^[1]^. Precultures of *Escherichia coli* HM0079^[2]^ carrying either the plasmid pTrc99a-*pmxA_1_*, pTrc99a-*pmxA_1_*-W454A, pTrc99a-*pmxA_1-2_*, pTrc99a-*pmxA_1-2_*-W454A or *E. coli* BL21 containing pOPINE-deoD^[3]^, which is coding for purine nucleoside phosphorylase (PNP), were prepared by inoculation of 3 mL LB media containing ampicillin (K029.4, Roth) or chloramphenicol (3886.2, Roth). Precultures were incubated at 37 °C at 180 rpm overnight in a rotary shaker. Main cultures were prepared by inoculation of 400 mL 2xYT media in 2 L flasks containing the appropriate antibiotics. The cultures were incubated at 37 °C at 250 rpm on a rotary shaker for approximately 4 h until they reached an OD_600_ of approximately 0.6. The cultures were cooled down to 18 °C and induced with 0.25 mM isopropyl-β-D-thiogalactoside (IPTG; BP1755-10, Fisher Scientific). Proteins were expressed overnight at 18 °C at 250 rpm. Cells were harvested by centrifugation and the supernatant was discarded. After resuspending the cell pellet in 30 mL lysis buffer (50 mM TRIS [pH 7.4], 500 mM NaCl, 20 mM imidazole, 2 mM TCEP), 100 μL protease inhibitor mix (APE-K1010, APExBio) were added, and cells were lysed by sonication while cooling on ice. The lysate was cleared by centrifugation at 19,000 g for 30 min at 4 °C and the supernatant was loaded onto a column packed with 2 mL of Ni-IDA suspension (1308.2, Roth) and equilibrated with lysis buffer. After washing the column twice with 20 mL of the lysis buffer, the target protein was eluted with 6 x 0.75 mL elution buffer (50 mM TRIS [pH 7.4], 500 mM NaCl, 300 mM imidazole, 2 mM TCEP). After pooling the protein-containing fractions, they were buffer exchanged with storage buffer (100 mM TRIS [pH 7.4], 500 mM NaCl, 2 mM TCEP), using Vivaspin 6 (Sartorius) filters with a cutoff of 10 kDa for PNP, 50 kDa for PmxA_1_ and PmxA_1_-W454A, or 100 kDa for PmxA_1-2_ and PmxA_1‑2_-W454A. Proteins were aliquoted in 10% glycerol and flash frozen in liquid nitrogen for storage at ‑80 °C. Protein concentrations were determined from the absorbance at 280 nm measured in Take3 plates on an Epoch2 microplate reader (Biotek) or a NanoDrop 2000 (Thermo) using calculated extinction coefficients (www.benchling.com). PNP was stored at a concentration of 500 µM in aliquots of 1.3 mg. The SDS-PAGE was prepared using a Bolt 4-12% Bis-Tris Plus gel (Thermo Fisher Scientific). The ladder used was a BlueEye Prestained Protein Marker 10-245 kDa (Jena Biosciences). The image was taken with an iBright 1500 (Invitrogen).

## MesG/hydroxylamine kinetics

Michaelis-Menten parameters of adenylation reactions were determined from kinetic data recorded with the MesG/hydroxylamine assay which was performed as described previously with minor modifications^[3,4]^. Reactions contained 50 mM TRIS (pH 7.6), 5 mM MgCl_2_, 100 μM 7‑ methylthioguanosine (MesG; PR3790-B100, Biosearch Technologies), 150 mM hydroxylamine (adjusted to pH 7.5-8 with NaOH), 5 mM ATP (A2383, Sigma), 1 mM TCEP, 0.4 U/mL inorganic pyrophosphatase (I1643, Sigma), 50 µM of PNP purified from *E. coli* in-house, the NRPS protein of interest (up to 2 µM), and a suitable amino acid substrate. The amino acids l-Ada, l-2-aminododecanoic acid and l-2-aminooctanoic acid were purchased from Fluorochem. In flat-bottom 384-well plates (781620, Brand), reactions were started in a total volume of 100 µL by addition of substrate. Amino acids were dissolved in DMSO and diluted to a final concentration of 0.1% DMSO. Reactions were started by adding the respective amino acid substrate solution and were measured against a control with 0.1% water. Then, the absorbance was followed at 355 nm on a Polarstar Omega microplate reader at 30 °C. Slopes for the background activity were recorded in wells containing buffer but not substrate and were subtracted. Each substrate concentration was measured as biological duplicate. Initial velocities were divided by the slope of a pyrophosphate calibration curve to obtain the pyrophosphate release rate. Michaelis-Menten parameters were extracted from the initial velocity data (v0/[E0]) through nonlinear regression in the R software package version 3.4.2.3.

In order to estimate the limit of detection (LOD), the background rate from 8 measurements was averaged (*v*_bck_ = 0.138 µM/min) and the standard deviation (SD) was determined (0.0165 µM/min). The LOD was calculated using the formula LOD = *v*_bck_ + 3×SD = 0.19 µM/min.

## Molecular dynamics simulation

The initial complex geometries were generated using Boltz-2^[5]^ using a truncated A­‑domain (Table S2). Molecular dynamics simulations were performed with GROMACS 2025.3^[6–11]^ using the CHARMM36-jul2022^[12,13]^ force field. Missing ligand topologies for Ada and Ada-AMP were generated with the CHARMM General Force Field (CGenFF, version 4.0)^[14]^. Ligand protonation states were assigned to reflect physiological pH. Atoms in the Boltz-2 ligand output were reordered and renamed, and hydrogen atoms were added to match the generated ligand topology.

Because the proper placement of the Mg^2+^ cofactor was not retained during Boltz-2 co-folding of the Ada-AMP complexes, its position was transferred from the 1amu crystal structure^[15]^. Side-chain rotamers of amino acid residues clashing with the inserted Mg^2+^ ion were adjusted using PyMOL’s mutagenesis tool to obtain low-strain conformations.

For the ATP/ligand complex, the two highest-scoring Boltz-2 structures were used as independent starting models as only they displayed correct placement of the Mg^2+^ ion in the active site. For the Ada-AMP system, one starting structure was used to generate five replicate trajectories with different initial velocities.

Each system was centered in a cubic solvent box with a minimum distance of 1.2 nm between the protein and the box edge and solvated with TIP3P^[16]^ water. Sodium and chloride ions were added to neutralize the net charge and yield a final salt concentration of 0.15 M. Structures were energy-minimized using the steepest descent algorithm, equilibrated under NVT and NPT conditions for 100 ps each, and subsequently simulated for 100 ns under NPT conditions. During equilibration, position restraints were applied to all complex heavy atoms including the ligand, with a force constant of 1000 kJ mol^-1^ nm^-2^.

All simulations were performed with periodic boundary conditions. A time step of 2 fs was used. Long-range electrostatic interactions were treated using the particle mesh Ewald method^[17]^. Van der Waals interactions were truncated at 1.2 nm using a force-switch function starting at 1.0 nm. Bonds involving hydrogen atoms were constrained using LINCS^[18]^. Temperature coupling was performed with the V-rescale thermostat^[18]^ at 300 K using separate coupling groups for the protein–ligand complex and the solvent/ions. Pressure coupling was applied isotropically using the Parrinello–Rahman barostat^[19]^.

## HAMA specificity profiling

Hydroxamate assays for adenylation specificity were performed as previously reported^[1]^. In brief, HAMA was conducted at room temperature in 100 μL volume containing 50 mM TRIS (pH 7.6), 5 mM MgCl_2_, 150 mM hydroxylamine (pH 7.5-8, adjusted with NaOH), 5 mM ATP (A2383, Sigma), 1 mM TCEP, and 0.2 mM proteinogenic amino acids with d-Val (Roth), d-Phe (Roth), deuterated l-Leu-d7, deuterated l-Phe-d5, deuterated l-Val-d8 and l-Ada (Fluorochem). Reactions were started by adding the NRPS protein to a final concentration of 0.1 µM. Samples were incubated for 30 min and 10 µL of the reaction was quenched in 95% ACN containing 0.1% formic acid (A117-50, Fisher Scientific), cooled down and centrifuged to remove the precipitated hydroxylamine. As control, heat denatured enzyme was used. Samples were measured as biological duplicates or triplicates as indicated. Samples were analyzed on a UPLC-MS/MS (Xevo TQ-S micro, Waters or TQS Quantis, Thermo Scientific) as described previously^[1]^ with the same mass transitions and MS conditions. The mass transition for the detection of Ada HA is 203.27 🡪 142.22. The cone voltage is 26 kV and the collision energy is 10 V.

## Synthesis

All chemicals for synthesis were purchased from Merck, Alfa Aesar, Acros Organics, Fluorochem, BLDpharm or TCI and used without further purification. The solvents were dried according to standard conditions if needed. The TLC-glass-plates DURASIL consisted of a 0.25 mm layer of silica 60 with Fluorescence indicator UV254. TLCs were checked under UV-light (254 nm or 365 nm) and stained with an aq. KMnO_4_- solution. ^1^H and ^13^C NMR spectra were measured on BRUKER Avance Neo Nanobay 300 spectrometers. The chemical shift of each signal was reported in ppm. As an internal standard, the residual ^1^H or ^13^C nuclei of the corresponding deuterated solvents were used (DMSO-d_6_, 2.50 ppm [^1^H-NMR], CDCl_3_, 7.27 ppm [^1^H-NMR], 77.16 ppm [^13^C-NMR]). For carbon spectra, a broadband decoupling was performed. High-resolution mass spectra (HRMS) were measured using ESI ionisation. The Thermo Q-Exactive plus device for ESI-mass spectra was coupled to a binary UHPLC system. All the IRs were measured using the Shimadzu IR-Affinity-1 (FTIR) device.

**Synthesis of l-2-aminodecanoic acid hydroxamate (Ada HA)**

**methyl (*S*)-2-((((9H-fluoren-9-yl)methoxy)carbonyl)amino)decanoate** **(2)**

Methyl ester **2** was prepared from commercially available (*S*)-*N*-Fmoc-2-octyl-L-glycine (**1**).

Trimethylsilyl chloride (206 µL, 1.62 mmol, 2.0 equiv.) was added to a solution of (*S*)-*N*-Fmoc-2-octyl-L-glycine **1** (333 mg, 0.81 mmol, 1.0 equiv.) in methanol (1.6 mL) at room temperature. The resulting mixture was stirred for 1 h and then concentrated under reduced pressure to give the desired methyl ester **2** as a colourless solid (340 mg, 0.803 mmol, 99% yield).

**^1^H NMR** (300 MHz, CDCl_3_) δ ppm 7.79 (d, *J* = 7.54 Hz, 2 H), 7.62 (m, 2 H), 7.43 (m, 2 H), 7.34 (m, 2 H), 5.31 (br d, *J* = 8.26 Hz, 1 H), 4.42 (m, 3 H), 4.26 (m, 1 H), 3.78 (s, 3 H), 1.85 (m, 1 H), 1.69 (m, 1 H), 1.29 (br s, 12 H), 0.91 (t, J = 6.55 Hz, 3 H). 173.21, 155.89, 143.95, 143.81, 141.33, 127.72, 127.08, 125.11, 120.00, 77.25, 67.00, 53.90, 52.37, 47.20, 32.68, 31.84, 29.36, 29.20, 25.19, 22.67, 14.13. **^13^C NMR** (75 MHz, CDCl_3_) δ ppm 173.31, 156.00, 144.05, 143.91, 141.44, 127.83, 127.18, 125.22, 120.11, 67.10, 54.01, 52.48, 47.31, 32.79, 31.94, 29.47, 29.31, 25.29, 22.77, 14.23. **HRMS** [ESI]: m/z calculated for C_26_H_33_NO_4_ [M+H]^+^ 424.2482, found 424.2486. **IR** (ATR): 3310 (w), 2951 (w), 2920 (b), 1740 (s), 1697 (s), 1539 (m), 1450 (m), 1273 (s), 741 (s), 482 (m).

**(*S*)-2-amino-*N*-hydroxydecanamide (3)**

Diethylamine (191 µL, 1.86 mmol, 10 equiv.) was added to a solution of the methyl ester **2** (78.7 mg, 0.186 mmol) in dichloromethane (4.6 mL) at 0 °C. The resulting mixture was allowed to reach room temperature and stirred overnight. Then, the reaction was concentrated under reduced pressure to give an oily residue, that was washed with diethylether (3 x 10 mL). The resulting mother liquor was concentrated under reduced pressure to give a yellow oil as a crude amino acid methyl ester (36.5 mg, 0.181 mmol, 98% yield), which was directly subjected to the next transformation.

A 6.0 M solution of KOH in dry methanol (75 µL, 0.447 mmol, 3.0 equiv.) was added to a 1.0 M solution of hydroxylamine hydrochloride in dry methanol (447 µL, 0.447 mmol, 3.0 equiv.) at 0 °C. The resulting mixture was kept at 0 °C for 15 min and was filtered through a Teflon 2.5 µm filter to remove the precipitate. The filtrate was then added to a solution of l-2-aminodecanoic acid methyl ester free base (30.0 mg, 0.149 mmol, 1 equiv.) in dry methanol (1 mL) and kept at -20 °C without stirring to facilitate crystallization. After 24 h, precipitate was filtered, washed with dry methanol, and dried under vacuum to afford the desired hydroxamate **3** as a colourless solid (4.0 mg, 0.019 mmol, 13% yield). The product was stored at -20 °C.

**^1^H NMR** (300 MHz, DMSO-d_6_) δ ppm 2.94 (m, 1 H), 1.20 (m, 14 H), 0.83 (m, 3 H). **HRMS** [ESI]: m/z calculated for C_10_H_22_N_2_O_2_ [M+H]^+^ 203.1754, found 203.1754, [M+Na]^+^ 225.1568, found 225.1572.

## Bacterial strains

*Paenibacillus polymyxa* DSM 365 was obtained from the German Collection of Microorganisms and Cell Culture (DSMZ), Braunschweig, Germany. *E. coli* Turbo™ (New England Biolabs) was used for plasmid cloning and *E. coli* S17-1 (ATCC 47055) was used for conjugation with *P. polymyxa*. Unless stated otherwise, all strains were cultivated in LB medium (10 g·L⁻¹ peptone, 5 g·L⁻¹ yeast extract, and 5 g·L⁻¹ NaCl). When required, the medium was supplemented with 50 µg·mL⁻¹ neomycin and 40 µg·mL⁻¹ polymyxin. *E. coli* strains were cultivated at 37 °C, whereas *P. polymyxa* was cultivated at 30 °C, unless otherwise indicated. For long-term storage, all strains were preserved in 30% (w/v) glycerol at −80 °C. All strains used in this study are listed in Table S3.

## Strain engineering

All plasmids for strain engineering were assembled using isothermal assembly and introduced into *E. coli* by heat shock transformation. DNA fragments were amplified using Q5 High-Fidelity DNA Polymerase (New England Biolabs). Plasmid DNA was isolated using the Thermo Fisher Scientific miniprep kit. Primers and plasmids used in this study are listed in Tables S4 and S5, respectively. Plasmids were transferred to *P. polymyxa* via conjugation as previously described^[20]^. Gene deletions and integrations were performed using the CRISPR–Cas9-based genome editing system developed for *P. polymyxa* by Rütering *et al*^[21]^. This system employs a guide RNA targeting the gene of interest to direct the Cas9 endonuclease to the cleavage site, thereby generating a double-strand break. Repair occurs through homologous recombination using flanking regions provided on the plasmid. For gene integration, the gene of interest was inserted between the homologous flanks. Genomic DNA was extracted using the Monarch gDNA Isolation Kit, and successful mutations were confirmed by sequencing (Figure S8).

## Genome sequencing of *P. polymyxa* DSM 292

High molecular weight DNA (HWD) was isolated with the MasterPure Complete DNA & RNA Purification Kit (Biozym, Hessisch Oldendorf, Germany) as recommended by the manufacturer. Quality of isolated DNA was initially checked by agarose gel electrophoresis and validated on an Agilent Bioanalyzer 2100 using an Agilent DNA 12000 Kit as recommended by the manufacturer (Agilent Technologies, Waldbronn, Germany). Concentration and purity of the isolated DNA was first checked with a Nanodrop ND-1000 (PeqLab Erlangen, Germany) and exact concentration was determined using the Qubit® dsDNA HS Assay Kit as recommended by the manufacturer (Life Technologies GmbH, Darmstadt, Germany). Illumina shotgun libraries were prepared using the Nextera XT DNA Sample Preparation Kit. To assess quality and size of the libraries, samples were run on an Agilent Bioanalyzer 2100 using an Agilent High Sensitivity DNA Kit as recommended by the manufacturer (Agilent Technologies, Waldbronn, Germany). Concentration of the libraries were determined using the Qubit® dsDNA HS Assay Kit as recommended by the manufacturer (Life Technologies GmbH, Darmstadt, Germany). Sequencing was performed on a MiSeq system with the reagent kit v3 with 600 cycles (Illumina, San Diego, CA, USA) as recommended by the manufacturer. For Nanopore sequencing 1.5 µg HWD was used for library preparation using the Ligation Sequencing Kit 1D (SQK-LSK114) as recommended by the manufacturer. Sequencing was performed for 72 h using a MinION device Mk1D (MinKNOW v25.03.9) and a SpotON Flow Cell R10.4.1 as recommended by the manufacturer (Oxford Nanopore Technologies) using MinKNOW software for sequencing and Dorado in high accuracy mode (v1.0.0) for basecalling.  Unicycler^[22]^ (v0.5.0) was used with default settings to perform a hybrid assembly. Annotation was performed with bakta v1.11.0^[23]^ and default settings in PGAP (v2025-05-06.build7983).^[24]^

## Fermentation of polymyxins

For optimization, fermentations were carried out using different media (Table S1) in baffled 100 mL flasks containing 20 mL media. The cultures were fermented at 30 °C at 180 rpm, using an Infors HT Multitron Pro orbital shaker (Infors HT) for 50 h. Samples were taken every 4 h. The cell count was determined using a Thoma counting chamber (Expert Medizinbedarf). For Ada-polymyxin production, fermentations were carried out using PBP Glucose media in baffled 1000 mL flasks containing 100 mL PBP Glucose media, supplemented with 1 mM l-2-aminodecanoic acid.

## Quantification of polymyxins

The polymyxin concentration was determined using a 1290 Infinity 2 (Agilent Technologies) UPLC system with a CORTECS UPLC C18 90Å, 1.6 µm, 2.1 mm x 50 mm column with a CORTECS C18 VanGuard Pre-column, 90 Å, 1.6 µm, 2.1 mm x 5 mm. Solvent A was H_2_O (HiPerSolv Chromanorm, VWR) with 0.1% formic acid (Fisher) and solvent B was MeCN (HiPerSolv Chromanorm, VWR). Samples were eluted with a gradient of 2% B to 80% over 2.5 min, followed by a 1.5 min wash and 1 min re-equilibration time. The UPLC was coupled to a TQS Quantis (Thermo Scientific). The double ionized polymyxin was detected after tuning with a polymyxin B sulfate standard (Roth). The spray voltage was set to 2,500 V, sheath gas to 15 arbitrary units, sweep gas to 2 arbitrary units, the ion transfer tube temperature to 350 °C and the vaporizer temperature to 350 °C. The mass transition for polymyxin B was m/z 602.38 (M+2H)^2+^ 🡪 100.95 for detecting the common 2,4-diaminobutyric acid fragment. The collision energy was set to 30 V and the RF Lens voltage to 200 V. Polymyxin P1 produced by DSM 365 was detected using the mass transition m/z 596.36 (M+2H)^2+^ 🡪100.953 and Ada-polymyxin using the mass transition m/z 607.4 (M+2H)^2+^ 🡪 100.953 for detecting the common 2,4-aminobutyric acid fragment. Polymyxins were quantified against a calibration curve of polymyxin B assuming identical detector response.

# Partial purification of polymyxin P1 from DSM 365

Fermentation was carried out in 5 baffled 2000 mL flasks containing 200 mL PBP_Glc_ media each. The cultures were fermented at 30 °C at 180 rpm, using an Infors HT Multitron Pro orbital shaker (Infors HT) for 50 h. The cells and residual solids were harvested by centrifugation at 5,000 g for 15 min. Supernatant was extracted with 1 volume ethylacetate at pH 2 in 5 batches using a separatory funnel. The aqueous phase contained most of the polymyxin and was frozen in liquid nitrogen and lyophilized overnight. The resulting powder was dissolved in the minimum amount of water and separated batchwise with a Chromabond C18 SPE column (Machery-Nagel) using methanol with 0.1% formic acid as eluent. The resulting factions were checked by LC-MS/MS for polymyxin P1 as described above. Fractions containing polymyxin were combined. The methanol was evaporated using a rotational evaporator (Heidolph) and diluted with water. The sample was lyophilized overnight. The resulting powder was dissolved in 5 mL water + 0.08% TFA. The sample was run over a Luna C18 5 µm 250 x 4.6 mm column attached to an Agilent 1240 HPLC with 500 µL injections. The gradient was 10% B to 25% B over 15 min, with A being water with 0.08% TFA and B being MeCN with 0.08% TFA. Fractions containing polymyxin P1 were collected, diluted with water and lyophilized overnight, resulting in 8.1 mg crude polymyxin P1 from 1 L fermentation culture, which was subjected to HRMS (Figure S1).

## HRMS measurement

The HRMS spectrum of Ada-polymyxin and polymyxin P1 was obtained using an Exactive Plus Orbitrap MS (Thermo Scientific), coupled to an Accucore Vanquish C18+ column (Thermo Scientific). The flow rate was 0.2 mL*min^-1^ with a gradient of 2% A to 98% B over 25 min, with A being water and B being acetonitrile. The column was flushed for 1 min with 98% B followed by 4 min of re-equilibration. The sheath gas flow rate was set to 45 arbitrary units, the aux gas flow rate to 10 arbitrary units, the sweep gas to 2 arbitrary units and the spray voltage to 3.5 kV. The capillary temperature was set to 250 °C, the S-lens RF level to 55.0 and the aux gas heater temperature to 400 °C.

## Disk diffusion assay

All *Paenibacillus* cultures were grown in PBP_Glc_ media. A culture with DSM 365::Δ*pmxA*, DSM 365::*pmxA*-W454A with 1 mM l-2-aminodecanoic acid and a culture with DSM 365::*pmxA*-W454A without l-2-aminodecanoic acid were prepared. The cultures were fermented at 30 °C at 180 rpm, using an Infors HT Multitron Pro orbital shaker (Infors HT) for 50 h. The cultures were spun down and the supernatant was extracted by SPE using a Chromabond C18 column (Waters), according to the manufacturer’s instructions. The polymyxins were eluted from the column using 90% methanol and 10% water containing 0.1% formic acid. The eluate was dried using a rotary evaporator and redissolved in 5 mL water. LB-agar was prepared in petri dishes with 92 mm diameter (Sarstedt). *E. coli* BL21 were grown in overnight cultures and adjusted to an OD = 0.5 using sterile 0.85% saline solution. The BL21 cultures were plated out on the LB-agar plates and dried under a sterile workbench for 10 min. 50 µL of the extracts were applied to a sterile disk. Water was used as solvent control and a 0.6 µM polymyxin B standard (Roth) was applied to a sterile disk as positive control. The disks were placed on the LB-agar plates and incubated for 6 h at 37 °C before diameters of inhibition zones were measured. Pictures were taken using an iBright 1500 (Invitrogen).

# Supplementary Tables

Table S1. Media tested for polymyxin P1 production with DSM 365.*

| Media | Titer [µM] | Composition | Reference |
| --- | --- | --- | --- |
| CN Glucose | 2.1 ± 0.8 | 21.1 g/L soy bean flour; 10 g/L soy bean oil; 0.1 g/L iron(II) sulfate; 25 g/L ammonium sulfate; 0.77 g/L potassium dihydrogenphosphate; 1 g/L calcium carbonate; 45 g/L glucose. | ^[25]^ |
| CN Starch | 32 ± 8 | 21.1 g/L soy bean flour; 10 g/L soy bean oil; 0.1 g/L iron(II) sulfate; 25 g/L ammonium sulfate; 0.77 g/L potassium dihydrogenphosphate; 1 g/L calcium carbonate; 5 g/L glucose; 40 g/L starch. | ^[25]^ |
| US | 22.1 ± 1.7 | 1 g/L calcium carbonate; 0.3 g/L potassium dihydrogenphosphate; 3 g/L ammonium sulfate; 12 g/L soy flour; 12 g/L wheat flour; 15 g/L glucose. | ^[26]^ |
| WO | 95 ± 4 | 20 g/L ammonium sulfate; 2 g/L dipotassium hydrogenphosphate; 0.5  g/L magnesium sulfate; 0.5 g/L sodium chloride; 0.01 g/L iron(II) sulfate; 5 g/L yeast extract; 20 g/L glucose; 30 g/L wheat starch. | ^[27]^ |
| PBP_Mal_ | 106 ± 9 | 60 g/L maltose; 3.61 g/L citric acid * H_2_O; 1 g/L potassium dihydrogenphosphate; 1.07 g/L ammonium sulfate; 1.62 g/L magnesium sulfate * 7 H_2_O; 5 g/L yeast extract; 10 g/L soy flour; 0.342 g/L calcium nitrate * 4 H_2_O; 5 mg/L thiamine HCl; 5 mg/L nicotinic acid; 0.2 mg/L riboflavin; 0.05 mg/L biotin; 1 mg/L calcium pantothenate; 5 mg/L pyridoxine HCl; 0.05 mg/L vitamin B12; 13 mg/L manganese(II) sulfate * H_2_O; 4.6 mg/L copper(II) sulfate; 2.8 mg/L sodium molybdate * 2 H_2_O; 15 mg/L iron(III) sulfate * H_2_O. | ^[28]^ |
| PBP_Sta_ | 110 ± 10 | 60 g/L starch; 3.61 g/L citric acid * H_2_O; 1 g/L potassium dihydrogenphosphate; 1.07 g/L ammonium sulfate; 1.62 g/L magnesium sulfate * 7 H_2_O; 5 g/L yeast extract; 10 g/L soy flour; 0.342 g/L calcium nitrate * 4 H_2_O; 5 mg/L thiamine HCl; 5 mg/L nicotinic acid; 0.2 mg/L riboflavin; 0.05 mg/L biotin; 1 mg/L calcium pantothenate; 5 mg/L pyridoxine HCl; 0.05 mg/L vitamin B12; 13 mg/L manganese(II) sulfate * H_2_O; 4.6 mg/L copper(II) sulfate; 2.8 mg/L sodium molybdate * 2 H_2_O; 15 mg/L iron(III) sulfate * H_2_O. | This study |
| PBP_Glc_ | 165 ± 3 | 60 g/L glucose; 3.61 g/L citric acid * H_2_O; 1 g/L potassium dihydrogenphosphate; 1.07 g/L ammonium sulfate; 1.62 g/L magnesium sulfate * 7 H_2_O; 5 g/L yeast extract; 10 g/L soy flour; 0.342 g/L calcium nitrate * 4 H_2_O; 5 mg/L thiamine HCl; 5 mg/L nicotinic acid; 0.2 mg/L riboflavin; 0.05 mg/L biotin; 1 mg/L calcium pantothenate; 5 mg/L pyridoxine HCl; 0.05 mg/L vitamin B12; 13 mg/L manganese(II) sulfate * H_2_O; 4.6 mg/L copper(II) sulfate; 2.8 mg/L sodium molybdate * 2 H_2_O; 15 mg/L iron(III) sulfate * H_2_O. | This study |
| Moppa | 90 ± 23 | 60 g/L maltose; 3.61 g/L citric acid * H_2_O; 3 g/L potassium dihydrogenphosphate; 3.21 g/L ammonium sulfate; 39 g/L MES buffer; 1.62 g/L magnesium sulfate * 7 H_2_O; 0.342 g/L calcium nitrate * 4 H_2_O; 0.06 mg/L nicotinic acid; 0.013 g/L biotin; 9 mg/L zinc sulfate * 7 H_2_O; 4 mg/L cobalt(II) sulfate * 7 H_2_O; 4 mg/L copper(II) sulfate * 5 H_2_O; 3 mg/L ammonium heptamolybdate * 4 H_2_O; 66 mg/L calcium chloride * 2 H_2_O; 16 mg/L manganese(II) chloride; 5 mg/L iron(II) chloride; 5 mg/L iron(III) chloride * 6 H_2_O | ^[28]^ |

* Polymyxin was measured with LC-MS/MS in the supernatant. 20 mL cultures were fermented in 100 mL baffled shaking flasks for 50 h at 30 °C at 180 rpm.

Table S2. Protein sequences used for Boltz-2 modelling.

| **Protein** | **Sequence** |
| --- | --- |
| PmxA_1_-A | SEDEKFQLLQSFNDTEKDYPRDRTIHQLVEEQAKRVPEATAVVFEGRRLSYAELNERANRLARTLRSVGVLPNQLVGLMVRRSLETVVGILAVLKAGGAYVPIDPEYPEERIRYILENSNAQLLLTQRHLLLQQVPFEGTVVALDDEQAYSDDGSNLEPASGPNDLAYVIYTSGTTGNPKGVMLEHRGLVSLKLMFADRLGITEHDRIVQFASLSFDASCWEVFKALYFGAALYIPTAETILDNRLFESYMNEHAITAAILPPTYSAYLNPDRLPSLTKLVTGGSAVSAEFVQQWKRKVHYFNAYGPTEASIVTTLWDADEEQPEGTVIPIGRPLANHRIFILDAHLQLVPPGVDGELCVAGVGLARGYLNHPELTAEKFVEHPFAPGERLYRTGDLARWLPDGNIEYLGRIDHQVKIRGFRIEIGEIEEQLLKIDSVQETIVIAREGKSGQELCAYLVAGR |
| PmxA_1_-A-W454A | SEDEKFQLLQSFNDTEKDYPRDRTIHQLVEEQAKRVPEATAVVFEGRRLSYAELNERANRLARTLRSVGVLPNQLVGLMVRRSLETVVGILAVLKAGGAYVPIDPEYPEERIRYILENSNAQLLLTQRHLLLQQVPFEGTVVALDDEQAYSDDGSNLEPASGPNDLAYVIYTSGTTGNPKGVMLEHRGLVSLKLMFADRLGITEHDRIVQFASLSFDASC**A**EVFKALYFGAALYIPTAETILDNRLFESYMNEHAITAAILPPTYSAYLNPDRLPSLTKLVTGGSAVSAEFVQQWKRKVHYFNAYGPTEASIVTTLWDADEEQPEGTVIPIGRPLANHRIFILDAHLQLVPPGVDGELCVAGVGLARGYLNHPELTAEKFVEHPFAPGERLYRTGDLARWLPDGNIEYLGRIDHQVKIRGFRIEIGEIEEQLLKIDSVQETIVIAREGKSGQELCAYLVAGR |

Table S3. Bacterial strains.

| **Name** | **Genotype** | **Source** | **Purpose** |
| --- | --- | --- | --- |
| *E. coli* HM0079 | *thrB1004 pro thi strA hsdS lacZ*ΔM15 (*F′ lacZ*ΔM15 *lacI*q *traD36 proA*⁺ *proB*⁺) *nrdD*::*sfp* Spcʳ | ^[2]^ | NRPS production |
| Stellar^TM^ Competent Cells | F⁻, *endA1*, *supE44*, *thi-1*, *recA1*, *relA1*, *gyrA96*, *phoA*, Φ80d *lacZ*ΔM15, Δ(*lacZYA-argF*)U169, Δ(*mrr-hsdRMS-mcrBC*), Δ*mcrA*, λ⁻ | Takara | Plasmid construction |
| DSM 292 | wildtype | DSMZ | Polymyxin production |
| DSM 365 | wildtype | AG Schmid | Polymyxin production |
| DSM 365::Δ*pmxA* | Δ*pmxA* | This study | Control strain |
| DSM 365::*pmxA*-W454A | *pmxA*-W454A | This study | Ada-polymyxin production |
| BL21(DE3) | *fhuA2* [*lon*] *ompT gal* (λ DE3) [*dcm*] ∆*hsdS* λ DE3 *=* λ sBamHIo ∆EcoRI-B int::(*lacI*::*P*_lacUV5_::*T7 gene1*) i21 ∆*nin5* | NEB | Disk diffusion assay |
| *E. coli* TOP10 | F-mcrAΔ(*mrr-hsdRMS-mcrBC*) Φ80*LacZ*ΔM15 Δ*Lac*X74 *rec*A1 *ara*D139 Δ(*araleu*) 7697*gal*U *gal*K *rps*L (StrR) *end*A1 *nup*G | Invitrogen | Plasmid construction |
| *E. coli* Turbo | F' *proA^+^B^+^ lacI^q^* ∆*lacZM15 / fhuA2* ∆(*lac-proAB) glnV galK16 galE15 R*(*zgb-210::Tn10*)TetS *end*A1 *thi*-1 ∆(*hsdS-mcrB*)5 | NEB | Plasmid construction |
| *E. coli* S17-1 | Conjugation strain; *recA pro hsdR* RP42Tc::Mu-Km::Tn7 integrated into the chromosome | ATCC 47055 | Conjugation |

Table S4. Primer list.

| **Name** | **Sequence** | **Used for** |
| --- | --- | --- |
| DSM292_pmxA_M6_full_A-C_f | CACAGGAAACAGACCATGTTTGAAAAAGAAACGTTGTT | pTrc99a-pmxA_1_ |
| DSM292_pmxA_M6_full_A-C_r | GTGATGGTGATGAGATCTCAGCTCGCCCACGGTAATTT |  |
| pmxA_M6-7_f1 | CACAGGAAACAGACCATGGCTTTTGAAAAAGAAACGTTGTTTTG | pTrc99a-pmxA_1_-W454A |
| pmxA_M6-7_r1 | GTGATGGTGATGAGATCTCAGCTCGGCAATCTTCGC |  |
| DSM292_pmxA_M6_frag2_f2 | AAAGCGCTCTATTTTGGCGCGGCTTTG |  |
| DSM292_pmxA_M6_frag1_W454A_r2 | CAAAATAGAGCGCTTTGAACACTTCCGCGCAGGA |  |
| pmxA_M6-7_f1 | CACAGGAAACAGACCATGGCTTTTGAAAAAGAAACGTTGTTTTG | pTrc99a-pmxA_1-2_ |
| pmxA_M6-7_r1 | GTGATGGTGATGAGATCTCAGCTCGGCAATCTTCGC |  |
| M6_W454_A_M7_oh_f1 | GAACTAGTGACGGAGGCGGAAAAG |  |
| M6_W454A_M7_oh_r1 | CTTTTCCGCCTCCGTCACTAGTTCCAGCTCGCCCACGGTAATTTC |  |
| pmxA_M6-7_f1 | CACAGGAAACAGACCATGGCTTTTGAAAAAGAAACGTTGTTTTG | pTrc99a-pmxA_1-2_-W454A |
| pmxA_M6-7_r1 | GTGATGGTGATGAGATCTCAGCTCGGCAATCTTCGC |  |
| M6_W454_A_M7_oh_f1 | GAACTAGTGACGGAGGCGGAAAAG |  |
| M6_W454A_M7_oh_r1 | CTTTTCCGCCTCCGTCACTAGTTCCAGCTCGCCCACGGTAATTTC |  |
| pCasPP_bb2_fw | TCTAGTGGCCAGGAACCGTAAAAAGG | Amplification of pCasPP backbone |
| sgRNA_rev | GCGTATCCCCTTTCAGATACTCGC | Amplification of pCasPP backbone |
| sgRNA_pmxA_dsm365_fw | GTATCTGAAAGGGGATACGCGAACTCGTTCGTCTGTACGGGTTTTAGAGCTAGAAATAG | Construction of pCasPP- ∆*pmxA*365 |
| oriT_rev | CTAGTCGGCGGGCTTGATGC | Construction of pCasPP- ∆*pmxA*365 |
| uhs_pmxA_dsm365_fw | GCATCAAGCCCGCCGACTAGCCGCAGTCGGATCTTTAC | Construction of pCasPP- ∆*pmxA*365 |
| uhs_pmxA_dsm365_rv | CTGGATCGTTCCCCTCTC | Construction of pCasPP- ∆*pmxA*365 |
| dhs_pmxA_dsm365_fw | AAGAGAGGGGAACGATCCAGTCCTACAAGCGCGCCTCC | Construction of pCasPP- ∆*pmxA*365 |
| dhs_pmxA_dsm365_rv | TACGGTTCCTGGCCACTAGACCAAGATGTCATGCGGCG | Construction of pCasPP- ∆*pmxA*365 |
| GR_sgRNA2_pmxA_dsm365_W454_fw | GTATCTGAAAGGGGATACGCCGTGCAGCAGTGGAAAGACAGTTTTAGAGCTAGAAATAG | Construction of pCasPP- *pmxA*DSM365-W454-A-2 |
| uhs_pmxA_dsm365_W454_rv | GAACATCTCCGCGCACGAC | Construction of pCasPP- *pmxA*DSM365-W454-A-2 |
| dhs_pmxA_dsm365_W454_fw | GTCGTGCGCGGAGATGTTC | Construction of pCasPP- *pmxA*DSM365-W454-A-2 |
| GR_dhs_pmxA_dsm365_sgRNA2_rv | AGTACTGTACATTGTCTTTCCAC | Construction of pCasPP- *pmxA*DSM365-W454-A-2 |
| GR_dhs_pmxA_dsm365_sgRNA2_fw | TGGAAAGACAAAGTACAGTAC | Construction of pCasPP- *pmxA*DSM365-W454-A-2 |
| dhs_pmxA_dsm365_W454_2_rv | TACGGTTCCTGGCCACTAGAAGTTCTTGCCCGCTTTTG | Construction of pCasPP- *pmxA*DSM365-W454-A-2 |
| ext_pmxA_dsm365_2_fw | CGTTCATGCCATACGGAG | Screening of *pmxA* deletion and point mutation |
| ext_pmxA_dsm365_2_rv | AGGCAGGTGCTGATGCTC | Screening of *pmxA* deletion and point mutation |
| seq_pmxA_dsm365_W454_fw | AGCGGTCCGAACGATCTG | Sequencing of the point mutation in *pmxA* |

*Table S5. Plasmids.*

| **Plasmids** | **Description** | **Source or reference** |
| --- | --- | --- |
| pCasPP | *P. polymyxa* CRISPR-Cas9 genome editing plasmid | ^[21]^ |
| pCasPP-∆*pmxA*365 | Deletion of polymyxin synthetase A | This study |
| pCasPP-*pmxA*DSM365-W454-A-2 | Point mutation in *pmxA* at position 454aa | This study |
| pOPINE-*deoD* | deoD gene to produce PNP | ^[3]^ |
| pTrc99a-pmxA_1_ | PmxA_1_ wildtype | This study |
| pTrc99a-pmxA_1-2_ | PmxA_1-2_ wildtype | This study |
| pTrc99a-pmxA_1_/W454A | PmxA_1_ W454A mutant | This study |
| pTrc99a-pmxA_1-2_/W454A | PmxA_1-2_ W454A mutant | This study |

# Supplementary Figures

**A)**

**B)**

**C)**

**[M+2H]^2+^**

**[M+Na]^+^**

**[M+H]^+^
+ 1.7 ppm**

**[M+H+Na]^2+^**

**[M+3H]^3+^**

Figure S1. A) Comparison of the polymyxin biosynthetic gene clusters from P. polymyxa DSM 365 (top) and P. polymyxa DSM 292 (bottom), made with antiSMASH.^[29]^ B) The chemical structures deduced from the AntiSMASH prediction assuming S-6-methyloctanoic acid as lipid tail, which agrees with the detected masses of the polymyxins. C) HRMS analysis of partially purified product from DSM 365 confirms the molecular formula of polymyxin P1 (calcd. [M+H]^+^: m/z 1191.7208, found m/z 1191.7229, Δ = 1.7 ppm).

Figure S2. SDS-PAGE, M: BlueEye Prestained Protein Marker (10-245 kDa). A) Proteins from DSM 292.1: PmxA_1-2_; 2: PmxA_1-2_-W454A; 3: PmxA_1_-W454A; 4: PmxA_1_. B) PmxA1-W454A from DSM 365. C) PmxA1 from DSM 365.

Figure S3. Michaelis-Menten kinetics of PmxA1 and PmxA1-W454A with l-2-aminooctanoic acid and l-2-aminododecanoic acid, respectively. Each substrate concentration was measured with two biological replicates.

Figure S4. Synthesis of 2-aminodecanoic acid hydroxamate (3) starting from (1) with (2) as intermediate product. Left NMR spectra are proton spectra the right spectra are carbon spectra. Top spectra belong to (2), bottom, spectra to (3)

Figure S5: (A) Top scored Boltz-2 generated initial structure of PmxA1-A, (grey) with ATP (orange), Ada (blue) and a magnesium ion (green) in the expected position, used for molecular dynamics simulation. (B) Two top-scored Boltz-2 generated initial structure of PmxA_1_-A-W454A (grey) with ATP (orange), Ada (blue) and a magnesium ion (green) in the expected position, used for molecular dynamics simulation. (C) Energy minimized structures of PmxA1-A (grey) with Ada-AMP (blue) and a magnesium ion (green), transferred from the 1amu PDB structure, in the expected position, used for molecular dynamics simulation. (D) Energy minimized structure of PmxA_1_-A-W454A (grey) with Ada-AMP (blue) and a magnesium ion (green), transferred from the 1amu PDB structure, in the expected position, used for molecular dynamics simulation.

Figure S6: (A) Minimum distance between catalytically critical residue Lys682 of PmxA1-A to the Ada carboxyl group over the two different 100 ns trajectories for each ATP/Ada and A-domain combination. The values were obtained between the heavy atoms of each group using the GROMACS mindist feature. (B) Minimum distance between catalytically critical residue Asp482 of PmxA_1_-A to the Ada amino group over the two different 100 ns trajectories for each ATP/Ada and A‑domain combination. The values were obtained between the heavy atoms of each group using the GROMACS mindist feature. (C) RMSD of Ada-AMP over 5 different 100 ns trajectories bound to PmxA_1_-A variants. (D) RMSD of Ada-AMP over 5 different 100 ns trajectories bound to PmxA_1_-A variants, when excluding the amino acid’s hydrophobic tail for RMSD calculation.

Figure S7. PmxA_1_-W454A (dark green) and GraA_5_ (light green) alignment. Specificity code residues are highlighted in purple (PmxA_1_-W454A) and teint (GraA_5_). Stachelhaus code for PmxA-A_1_-W454A is DAAIVAIVL, and for GraA_5_ DVGVVGVLA.

Figure S8. Sequencing of DSM 365::pmxA-W454A with Sanger sequencing to verify the cloning success.

Figure S9. Fragmentation pattern of a polymyxin B standard. The resulting 2,4-l-Dab fragment can appear 5 times per polymyxin B molecule. Daughter ion marked in red.

Figure S10. HRMS spectrum of Ada-polymyxin. At a retention time of 10.49 min, the exact mass of 405.271 [M+3H]^3+^ is found in the culture supernatant with an error of 0 ppm.

Figure S11. Comparison of DSM 365::pmxA-W454A (left) and DSM 365::ΔpmxA (right) after 50 h of fermentation. Left culture visually appears lysed.

Figure S12. Disk diffusion assay. 1: solvent control (water); 2: extract of an Ada deficient culture of DSM 365::pmxA-W454A; 3: culture extract DSM 365::ΔpmxA; 4: positive control polymyxin B sulfate (0.6 µM); 5: culture extract of DSM 365::pmxA-W454A. Three replicates were prepared from separate cultures.

# Bibliography

[1]	A. Stanišić, A. Hüsken, H. Kries. *Chem. Sci.* **2019**, *10*, 10395–10399.

[2]	S. Gruenewald, H. D. Mootz, P. Stehmeier, T. Stachelhaus. *Appl. Environ. Microbiol.* **2004**, *70*, 3282–3291.

[3]	M. Müll, F. Pourmasoumi, L. Wehrhan, O. Nosovska, P. Stephan, H. Zeihe, I. Vilotijevic, B. G. Keller, H. Kries. *RSC Chem. Biol.* **2023**, *4*, 692–697.

[4]	D. J. Wilson, C. C. Aldrich. *Anal. Biochem.* **2010**, *404*, 56–63.

[5]	S. Passaro, G. Corso, J. Wohlwend, M. Reveiz, S. Thaler, V. R. Somnath, N. Getz, T. Portnoi, J. Roy, H. Stark, D. Kwabi-Addo, D. Beaini, T. Jaakkola, R. Barzilay., **2025**, Molecular Biology preprint, DOI: 10.1101/2025.06.14.659707.

[6]	M. Abraham, A. Alekseenko, B. Andrews, V. Basov, P. Bauer, H. Bird, E. Briand, A. Brown, M. Doijade, G. Fiorin, S. Fleischmann, S. Gorelov, G. Gouaillardet, A. Gray, M. E. Irrgang, F. Jalalypour, P. Johansson, C. Kutzner, G. Łazarski, J. A. Lemkul, M. Lundborg, P. Merz, V. Miletić, D. Morozov, L. Müllender, J. Nabet, S. Páll, A. Pasquadibisceglie, M. Pellegrino, N. Piasentin, D. Rapetti, M. U. Sadiq, H. Santuz, R. Schulz, M. Shirts, T. Shugaeva, A. Shvetsov, P. Turner, A. Villa, S. Wingbermühle, B. Hess, E. Lindahl. **2025**, DOI 10.5281/ZENODO.16992621.

[7]	M. J. Abraham, T. Murtola, R. Schulz, S. Páll, J. C. Smith, B. Hess, E. Lindahl. *SoftwareX* **2015**, *1–2*, 19–25.

[8]	B. Hess, C. Kutzner, D. Van Der Spoel, E. Lindahl. *J. Chem. Theory Comput.* **2008**, *4*, 435–447.

[9]	D. Van Der Spoel, E. Lindahl, B. Hess, G. Groenhof, A. E. Mark, H. J. C. Berendsen. *J. Comput. Chem.* **2005**, *26*, 1701–1718.

[10]	E. Lindahl, B. Hess, D. Van Der Spoel. *J. Mol. Model.* **2001**, *7*, 306–317.

[11]	H. J. C. Berendsen, D. Van Der Spoel, R. Van Drunen. *Comput. Phys. Commun.* **1995**, *91*, 43–56.

[12]	R. B. Best, X. Zhu, J. Shim, P. E. M. Lopes, J. Mittal, M. Feig, A. D. MacKerell. *J. Chem. Theory Comput.* **2012**, *8*, 3257–3273.

[13]	J. Huang, A. D. MacKerell. *J. Comput. Chem.* **2013**, *34*, 2135–2145.

[14]	K. Vanommeslaeghe, E. Hatcher, C. Acharya, S. Kundu, S. Zhong, J. Shim, E. Darian, O. Guvench, P. Lopes, I. Vorobyov, A. D. Mackerell. *J. Comput. Chem.* **2010**, *31*, 671–690.

[15]	E. Conti. *EMBO J.* **1997**, *16*, 4174–4183.

[16]	W. L. Jorgensen, J. Chandrasekhar, J. D. Madura, R. W. Impey, M. L. Klein. *J. Chem. Phys.* **1983**, *79*, 926–935.

[17]	U. Essmann, L. Perera, M. L. Berkowitz, T. Darden, H. Lee, L. G. Pedersen. *J. Chem. Phys.* **1995**, *103*, 8577–8593.

[18]	B. Hess, H. Bekker, H. J. C. Berendsen, J. G. E. M. Fraaije. *J. Comput. Chem.* **1997**, *18*, 1463–1472.

[19]	M. Parrinello, A. Rahman. *J. Appl. Phys.* **1981**, *52*, 7182–7190.

[20]	G. Ravagnan, J. Lesemann, M.-F. Müller, A. Poehlein, R. Daniel, S. Noack, J. Kabisch, J. Schmid. *Front. Bioeng. Biotechnol.* **2024**, *12*, DOI 10.3389/fbioe.2024.1378873.

[21]	M. Rütering, B. F. Cress, M. Schilling, B. Rühmann, M. A. G. Koffas, V. Sieber, J. Schmid. *Synth. Biol.* **2017**, *2*, ysx007.

[22]	R. R. Wick, L. M. Judd, C. L. Gorrie, K. E. Holt. *PLOS Comput. Biol.* **2017**, *13*, e1005595.

[23]	O. Schwengers, L. Jelonek, M. A. Dieckmann, S. Beyvers, J. Blom, A. Goesmann. *Microb. Genomics* **2021**, *7*, DOI 10.1099/mgen.0.000685.

[24]	W. Li, K. R. O’Neill, D. H. Haft, M. DiCuccio, V. Chetvernin, A. Badretdin, G. Coulouris, F. Chitsaz, M. K. Derbyshire, A. S. Durkin, N. R. Gonzales, M. Gwadz, C. J. Lanczycki, J. S. Song, N. Thanki, J. Wang, R. A. Yamashita, M. Yang, C. Zheng, A. Marchler-Bauer, F. Thibaud-Nissen. *Nucleic Acids Res.* **2021**, *49*, D1020–D1028.

[25]	Z. Yu, Z. Sun, J. Yin, J. Qiu. *BioMed Res. Int.* **2018**, *2018*, 1–10.

[26]	I. Varga, K. Vrúblová, L. Kovácová, J. Kluková., *Process for the Preparation of Polymyxin B Employing (Paeni)Bacillus Polymyxa*, **2012**, US008119371B2.

[27]	V. Rangaswamy, G. Balu, A. Hiremath., *Process for the Production and Purification of Polymyxin B Sulfate*, **2010**, WO 2010/058427 A2.

[28]	J. Goldmanns, G. A. Röhling, M. K. Lipa, T. Scholand, A. Deitert, T. May, E. P. Haas, M. Boy, A. Herold, J. Büchs. *BMC Biotechnol.* **2023**, *23*, DOI 10.1186/s12896-023-00793-7.

[29]	K. Blin, S. Shaw, L. Vader, J. Szenei, Z. L. Reitz, H. E. Augustijn, J. D. D. Cediel-Becerra, V. de Crécy-Lagard, R. A. Koetsier, S. E. Williams, P. Cruz-Morales, S. Wongwas, A. E. Segurado Luchsinger, F. Biermann, A. Korenskaia, M. M. Zdouc, D. Meijer, B. R. Terlouw, J. J. J. van der Hooft, N. Ziemert, E. J. N. Helfrich, J. Masschelein, C. Corre, M. G. Chevrette, G. P. van Wezel, M. H. Medema, T. Weber. *Nucleic Acids Res.* **2025**, *53*, W32–W38.
